# Characterizing Cancer‐Related Cognitive Impairment Among Adolescent and Young Adult Cancer Survivors: Comparison to Individuals Without Cancer

**DOI:** 10.1002/cam4.71363

**Published:** 2025-11-05

**Authors:** Brent J. Small, Damon Reed, Danielle Tometich, Amarilis Nieves‐Lopez, Nathaly Irizarry‐Arroyo, Hannah Decosta, Laura B. Oswald, Heather S. L. Jim

**Affiliations:** ^1^ School of Nursing University of North Carolina at Chapel Hill Chapel Hill North Carolina USA; ^2^ Cancer Prevention and Control Program, UNC Lineberger Comprehensive Cancer Center Chapel Hill North Carolina USA; ^3^ Division of Pediatric Solid Tumors Memorial Sloan Kettering Cancer Center New York New York USA; ^4^ College of Nursing University of South Florida Tampa Florida USA; ^5^ Department of Health Outcomes and Behavior Moffitt Cancer Center Tampa Florida USA

**Keywords:** adolescent and young adult cancer survivors, cancer‐related cognitive impairment, patient‐reported outcomes, quality of life

## Abstract

**Background:**

Cancer‐related cognitive impairment (CRCI) has received little attention among adolescent and young adult (AYA; 18–39 years of age) cancer survivors. The goal of this study was to evaluate differences in objective and subjective cognitive performance and quality of life between AYA survivors and individuals without a cancer history.

**Methods:**

AYA cancer survivors were recruited from clinic databases, and noncancer controls were identified through Research Match. Objective cognitive performance was measured using the Cambridge Neuropsychological Test Connect Automated Battery which assessed attention, executive functioning, and memory. Participants completed questionnaires on PROMIS measures of subjective cognition and quality of life (QOL). Mean differences were evaluated using ANCOVAs, independent of age and education.

**Results:**

The sample included 88 AYA survivors and 96 noncancer controls (NC). The AYA survivors were older (M_AYA_ = 34.1, M_NC_ = 28.8, *p* < 0.001) and less likely to have a college education (% _AYA_ = 67.0, % _NC_ = 79.2, *p* = 0.063). The AYA group averaged almost 4 years since the end of treatment, and the majority were lymphoma/leukemia survivors (27%), followed by breast cancer survivors (14%). On objective tests of cognition, there was a trend (*p* = 0.061) for lower performance in attention among AYA survivors (*d* = 0.11), but no differences were observed for executive functioning (*d* = 0.03) or memory (*d* = 0.03). AYA survivors rated their cognition as worse than the NC group on PROMIS measures of subjective cognitive function (*d* = 0.69) and cognitive function abilities (*d* = 0.57; *p* < 0.001). Significant group differences were seen on multiple PROMIS QOL measures (*d* ranging from 0.32 to 0.77), all in favor of the NC group (*p* < 0.05).

**Conclusions:**

Results indicated that AYA survivors performed similarly on measures of objective cognitive performance, despite reporting worse subjective cognitive functioning and poorer quality of life. Further research is needed to address the disconnect between objective and subjective cognitive performance among AYA cancer survivors.

## 1 Background

There have been dramatic increases in the number of adolescent and young adult cancer survivors (AYA; 15–39 years) [1, 2], due, in part, to 5‐year survival rates over 85% and estimates suggest that there are over 2 million AYA survivors in the United States [3]. Cancer‐related cognitive impairment (CRCI) is common and distressing among people with cancer [4, 5, 6], yet relatively empirical work has been reported in the AYA population [7, 8]. A recent review noted that “CRCI has not been comprehensively characterized among AYAs” [9]. CRCI may have long‐term consequences on educational pursuits, work productivity, and unemployment [10], burdens among AYA survivors [11] that may exacerbate issues with the financial toxicity of cancer treatment in this population [12].

For AYA cancer survivors, the diagnosis and treatment of cancer coincide with the development and peak of various cognitive abilities, making them particularly vulnerable to CRCI [13, 14]. Among AYA cancer survivors, studies have reported cognitive deficits prior to the initiation of treatment [15], as well as self‐reports of difficulty remembering and concentrating more than 10 years after diagnosis [16]. A recent qualitative study nicely illustrates the challenges that AYA survivors report concerning their cognitive performance [17]. For example, one AYA survivor stated that “going to the grocery store, I will completely forget if I don't write down a distinct list; I will absolutely forget what we need” and another mentioned “when I'm fatigued, I just don't have the space to do anything… I just feel like I don't have the bandwidth to do it.” Finally, one cancer survivor expressed frustration with remembering names of people that they just met and said “I can't remember that we talked for an hour last week like they said. They're just erased to me, they're gone.” These testimonials reflect how changes to cognitive performance can impact everyday life, as well as the ability to maintain social relationships.

A gap in current work on CRCI among AYA cancer survivors is that most studies have evaluated self‐reports of cognitive functioning, rather than relying on evaluating objective functioning using standard neuropsychological indicators. Among cancer survivors, there is often a disconnect whereby a high prevalence of cognitive impairment from subjective measures of CRCI contrasts against more subtle deficits from objective measures of performance [18, 19]. Further, self‐reports of cognitive performance are related to depressed mood, anxiety or fatigue among cancer survivors, whereas objective measures often show little association with quality‐of‐life indicators [20]. Existing evidence among AYA survivors relies almost exclusively upon self‐reports of cognitive functioning, which may portray an incomplete picture of CRCI in this population.

In order to evaluate how individuals with a history of cancer differed from persons without cancer, we examined differences between AYA cancer survivors and persons without a history of cancer on subjective and objective measures of cognitive performance, as well as a diverse set of quality‐of‐life indicators. Our specific goals were to: (1) evaluate differences on objective measures of cognitive performance, focusing on measures of attention, executive functioning, and memory, domains of cognitive performance that have been shown to be vulnerable in adult populations, (2) evaluate differences in self‐report measures of subjective cognitive performance, and (3) evaluate differences in QOL measures, including anxiety, depressed mood, fatigue, sleep, and physical function.

## 2 Methods

### 2.1 Participants and Procedures

Potential participants who had been diagnosed with cancer were identified from the AYA survivorship program at the Moffitt Cancer Center. An introductory email from the AYA survivorship program with a link to a survey to confirm eligibility was sent to potential participants. Persons were provided with a secure website link to login. Eligible participants: (a) were between the ages of 18–45; (b) had been diagnosed with noncentral nervous system cancer between the ages of 18–39 and had completed curative treatment; (c) had no documented psychiatric or neurological disorders that would interfere with study participation (e.g., dementia, psychosis); (d) were capable of speaking and reading standard English; and (e) had no recurrence of cancer.

Potential noncancer control participants were recruited through Research Match. Persons were provided with a secure website link to login. Eligible participants: (a) were between the ages of 18–45; (b) had no history of cancer, other than nonmelanoma skin cancer; (c) had no documented psychiatric or neurological disorders that would interfere with study participation (e.g., dementia, psychosis); and (d) were capable of speaking and reading standard English.

For both groups of participants, an information sheet was presented with details regarding the study, and participants provided online consent. Participants were offered monetary incentives of $25 to complete the online questionnaires and $25 for the online neuropsychological assessment. This study was approved by Advarra Inc. Institutional Review Board (Pro00058328).

### 2.2 Measures

*Objective cognitive performance*. This domain was evaluated using the internet‐based Cambridge Neuropsychological Test Connect Automated Battery (CANTAB), a well‐validated battery of tests based on neuropsychology and neuroscience. The battery is administered in a semiautomated manner via the web, which is automatically scored upon completion [21, 22]. CANTAB has been used extensively in studies of CRCI [23, 24, 25] and minimizes burden by not requiring persons to come for lengthy paper‐and‐pencil neuropsychological assessments. The selected battery focused on domains of cognition that have been assessed in cancer survivors in our previous work [26, 27, 28]. Within the domains of attention, executive functioning, and memory, we identified relevant cognitive instruments and used the recommended outcomes according to published guidelines ([29]; see Table S1).

### 2.3 Subjective Cognitive Performance

This domain was assessed using the PROMIS Cognitive Functioning 8a and Cognitive Abilities 8a measures [30].

### 2.4 Quality of Life

Measures taken from the PROMIS set of instruments were included due to their excellent psychometric properties and normative database [31]. The PROMIS Depression Short Form 8a [32] was used to assess depressed mood during the previous 7 days. Anxiety was assessed using the PROMIS Anxiety 8a scale. Fatigue was assessed using the PROMIS Fatigue 8a scale, which evaluates perceptions of fatigue severity and interference in the past 7 days [33, 34]. Finally, sleep quality over the past 7 days was assessed with the PROMIS Sleep Disturbance 8a and PROMIS Sleep Related Impairment 8a scales [35].

### 2.5 Statistical Analyses

Descriptive statistics were used to summarize demographic and clinical characteristics, with one‐way ANOVAs and chi‐square tests being used to determine group differences and whether covariates were necessary for subsequent analyses. Given the large number of subtests generated by the CANTAB assessment, the groups were compared on aggregate measures of attention, executive functioning, and memory performance using meta‐analytic summaries that were based upon covariate‐adjusted means and standard deviations. ANCOVAs, with age and education as covariates, were conducted to evaluate group differences in the subjective cognitive and quality of life outcomes. In all cases, the magnitude of the group differences was scaled as standardized mean differences (i.e., Cohen's d). Given the sample size examined here, we have 80% power at alpha of 0.05 and a two‐tailed test of significance to detect differences that are at least 0.42 SD units between AYA survivors and controls.

## 3 Results

### 3.1 Demographic Characteristics

The sample consisted of 88 AYA cancer survivors (AYA) and 96 participants with no history of cancer (NC). Table 1 shows the demographic characteristics of the sample. The cancer survivors were significantly older (*p* < 0.001), and there was a trend for the NC group having a higher percentage of college graduates (*p* = 0.063). No significant differences were observed for sex (*p* = 0.215) or race (*p* = 0.189). Among the AYA cancer survivors, on average, they were 30.3 years old at diagnosis and averaged 3.9 years since the end of treatment. The greatest percentage of diagnoses included 27% of participants with lymphoma/leukemia, 14% with breast cancer, and 12% each with testicular cancer or sarcoma.

**TABLE 1:** Descriptive characteristics of the sample.

N		AYA survivors	Noncancer controls	*p*
		88	96	
Age	M	34.1	28.8	< 0.001
	SD	6.0	6.1	
Sex (% Female)		67.8	76.0	0.215
Race (% White)		80.2	71.9	0.189
Education (% College)		67.0	79.2	0.063
Age at diagnosis	M	30.26	—	
	SD	6.15	—	
Years since diagnosis	M	3.88	—	
	SD	3.53	—	
Primary Cancer Site, No. (%)^a^				
	Leukemia/Lymphoma	26 (27%)		
	Testicular	12 (12%)		
	Breast	14 (14%)		
	Sarcoma	12 (12%)		
	Gastrointestinal	6 (6%)		
	Thyroid	6 (6%)		
	Skin/Melanoma	3 (3%)		
	Gynecologic	2 (2%)		
	Kidney	2 (2%)		
	Bone and Connective	2 (2%)		
	Head and Neck	1 (1%)		

^a^ Categories are not mutually exclusive, as some persons had multiple cancer diagnoses.

### 3.2 Group Differences in Objective Cognitive Performance

The means and standard errors for each of the recommended outcomes of the CANTAB cognitive domains by AYA and NC groups are shown in Table S2. Across the measures of attention, there was a trend for the AYA group to perform significantly worse on the aggregate measure (*d* = −0.11, SE = 0.06, *p* = 0.061), with only one of the subtests, the delayed match to sample for the 4‐box condition, exhibiting a trend (*p* = 0.069) toward the AYA group performing more poorly. For the executive functioning domain, the aggregate difference effect size was not statistically significant (*d* = −0.03, SE = 0.19, *p* = 0.879), and neither of the individual outcomes were significant. Finally, for memory, the aggregate effect size was not statistically significant (*d* = −0.03, SE = 0.08, *p* = 0.675), and only one of the seven outcomes was statistically significant, with the AYA group performing more poorly on the Paired Associates Learning first attempt memory score.

### 3.3 Group Differences in Subjective Cognitive Performance and Quality of Life

**FIGURE 1:**
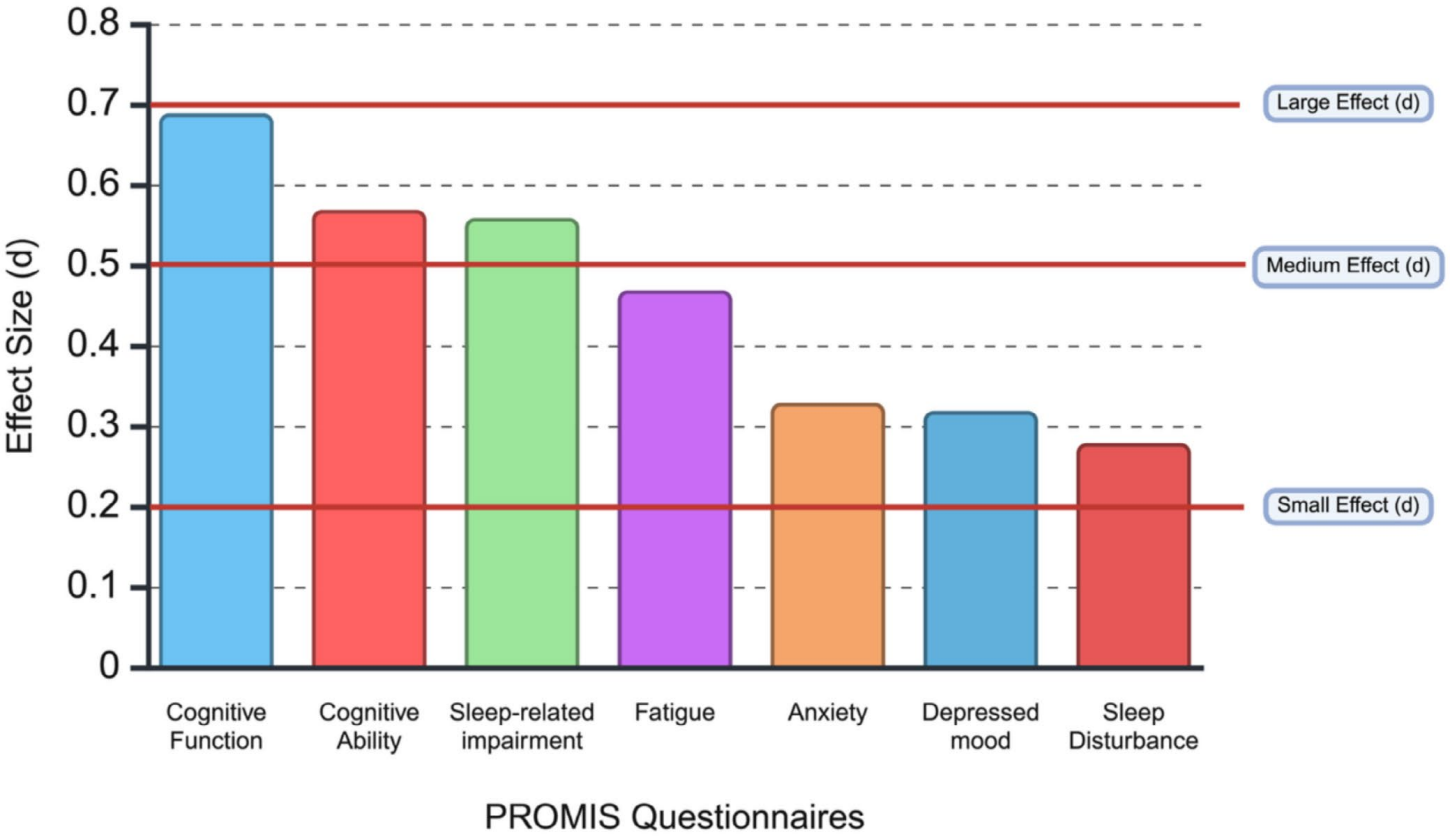
Effect sizes for worse functioning among AYA survivors, compared to controls (created in https://BioRender.com).

Table 2 and Figure 1 display the results from the PROMIS self‐reported measures of cognitive performance and quality of life. Significant group differences were observed for both cognitive function (F(1, 179) = 19.91, *p* < 0.001) and cognitive function abilities (F(1, 179) = 13.47, *p* < 0.001), with AYA survivors reporting worse cognitive performance as compared to noncancer controls. Significant group differences were also seen for most measures of quality of life, including anxiety (F(1, 179) = 4.61, *p* = 0.033), depressed mood (F(1, 179) = 4.18, *p* = 0.042), fatigue (F(1, 179) = 9.02, *p* = 0.003), and sleep‐related impairment (F(1, 179) = 12.96, *p* < 0.001). There was a trend for differences in sleep disturbance (F(1, 179) = 3.17, *p* = 0.077). In all cases, the AYA group rated their quality of life as worse than the noncancer controls.

**TABLE 2:** Self‐reported PROMIS measures Group differences.

N		AYA Survivors	Non‐Cancer Controls	Effect Size (d)	*p*
		87	96		
Self‐reported Cognition					
Cognitive function	M	44.78	51.57	0.69	< 0.001
	SD	9.81	9.77		
Cognitive function abilities	M	45.16	51.72	0.57	< 0.001
	SD	11.51	11.46		
Quality of Life					
Anxiety	M	56.18	53.11	0.33	0.033
	SD	9.2	9.16		
Depressed mood	M	51.16	48.16	0.32	0.042
	SD	9.46	9.43		
Fatigue	M	54.37	49.88	0.47	0.003
	SD	9.64	9.6		
Sleep disturbance	M	51.24	48.84	0.28	0.077
	SD	8.69	8.66		
Sleep‐related	M	54.13	49.6	0.56	< 0.001
impairment	SD	8.1	8.08		

*Note:* Means and SD reflect age and education‐adjusted T‐scores.

## 4 Discussion

In this study, we comprehensively evaluated CRCI among AYA cancer survivors on multiple measures of objective and subjective cognitive performance. The results indicated that there was a trend for AYA survivors to perform more poorly in attention, but no differences were observed for executive functioning or memory. By contrast, statistically significant and large differences were seen on subjective cognitive measures, with the AYA group reporting worse performance, relative to controls. Finally, our results indicated worse self‐reported functioning across several quality‐of‐life domains, including anxiety, depressed mood, fatigue, sleep‐related impairment, and physical function.

In the current study, our results indicated that measures of objective cognitive performance were generally similar in the AYA and NC groups. These results are at odds with a recent study describing pretreatment differences between AYA survivors and healthy controls [15]. In this study, over 20% of the AYA sample exhibited impairment, defined as 1.5 SD below the mean of the healthy control sample, and for several domains, the impairment was significantly higher than the control sample. However, when mean‐level performance was compared between the groups, no significant differences were observed for memory, executive functioning, or attention by Chan and colleagues, and these results were consistent with the patterns of effects that we observed here. A caveat of this comparison is that the period of assessment differed between our work (posttreatment) and research by Chan and colleagues (pretreatment).

The results of the current study contribute to the limited body of literature that has documented worse self‐reported cognitive performance among AYA cancer survivors [9]. In the current study, we found statistically significant and large effect sizes for the comparisons between AYA survivors and controls. The presence of significant deficits in CRCI when measured using self‐report instruments but modest differences on objective measures is a consistent observation among studies of CRCI [19]. Ferguson and colleagues suggested that more effortful processing among cancer survivors is needed to achieve the same cognitive performance, which may explain the discrepancy between objective and subjective cognition [36]. Costa and Fardell argued that there may be several reasons for this disconnect, suggesting that self‐report measures may be more reflective of CRCI in daily life, as compared to objective assessments which capture cognition under optimal, controlled situations [18]. In our own work, we have increasingly relied upon measuring objective cognitive functioning with a combination of standard neuropsychological instruments, as well as evaluating performance in daily life by administering tests multiple times a day using smartphones. We have observed considerable variability with and across days among breast cancer survivors [37], differences in memory lapses between cancer survivors and healthy controls [38], as well as subjective memory lapses predicting objective cognitive performance among cancer survivors [20]. Augmenting traditional measures of subjective and objective cognitive performance, with indicators of functioning in daily life may allow us to more thoroughly characterize CRCI among AYA cancer survivors.

The results of the current study also indicated that AYA cancer survivors reported worse anxiety, fatigue, sleep‐related impairment, and physical functioning. A recent meta‐analysis demonstrated that a third of AYA survivors exhibited psychological distress or anxiety, and a quarter were impacted by depressed mood [39]. Our results contribute to this literature and highlight the psychological needs among AYA survivors, even many years after they have been diagnosed with cancer. Identifying the origins of these lingering impacts on the quality of life, as well as potential interventions to improve functioning, will be critical in future studies.

### 4.1 Clinical Implications

Our results demonstrate that AYA cancer survivors report long‐term disruptions to cognitive performance and quality of life. This research suggests that administering these kinds of measures among AYA survivors will allow clinicians to evaluate domains of strength and weakness among the persons that they treat. Attention to the psychosocial needs of this group, even many years after diagnosis and end of treatment, is critical, in terms of making survivors aware of the potential for CRCI, as well as for clinicians to address the concerns that survivors report.

### 4.2 Strengths and Limitations

There are several strengths and potential weaknesses with the current results. The strength of this paper includes the inclusion of validated measures of objective and subjective cognitive performance, as well as quality of life. In previous research [16], a single item has been used to measure subjective cognitive performance, whereas we have used a psychometrically derived instrument that has increasingly wide application for CRCI [19]. Our battery of objective cognitive functioning also included validated instruments that reflect component processes from neuropsychology and neuroscience. Potential limitations of the current paper also exist, including the lack of pretreatment assessment of cognitive performance, as well as the heterogeneity of cancer types. Further, we were also unable to evaluate the potential impact of the type of cancer treatment on cognitive performance. Future research should focus on a smaller number of types of cancer, as well as examine how treatment modality impacts the severity of cognitive problems that individuals report.

## 5 Conclusion

In the current study, our results provide additional evidence for the vulnerability of AYA cancer survivors to experience long‐term impacts on CRCI, as well as QOL more broadly. Our research demonstrates clear differences between AYA survivors and controls in terms of their perceptions of cognitive abilities and quality of life. The presence of CRCI in this population may have implications for return to school or employment in this group. For example, existing research reported that AYA survivors were less likely to be employed 1 year following diagnosis, and this was related to diagnosis and treatment‐related factors, but no information was provided regarding QOL or CRCI [40]. Consideration of these critical and potentially modifiable characteristics should receive greater attention among AYA cancer survivors.

## Author Contributions

Brent J. Small, PhD: conceptualization, data curation, formal analysis, funding acquisition, writing – original draft. Damon Reed, MD: conceptualization, investigation, funding acquisition, writing – review and editing. Danielle Tometich, PhD: conceptualization, investigation, writing – review and editing. Amarilis Nieves‐Lopez: project administration, writing – review and editing; Nathaly Irizarry‐Arroyo: project administration, writing – review and editing; Hannah Decosta: project administration, writing – review and editing; Laura B. Oswald, PhD: conceptualization, writing – review and editing; Heather S.L. Jim, PhD: conceptualization, data curation, formal analysis, funding acquisition, writing – original draft.

## Ethics Statement

The study was approved by the Advarra Institutional Review Board (Pro00058328), and participants provided electronic consent.

## Conflicts of Interest

Dr. Reed: Springworks; Eisai: Data Safety monitoring board; Dr. Jim: Consultant for SBR Biosciences, grant funding from Kite Pharma. The other authors have no conflicts of interest to disclose.

## Supporting information


**Table S1:** Description of the CANTAB test domains and individual tests.
**Table S2:** Group differences in CANTAB objective cognitive performance.

## Data Availability

The data that support the findings of this study are available on request from the corresponding author. The data are not publicly available due to privacy or ethical restrictions.

## References

[1] Stat Bite: Cancer Among Adolescents and Young Adults,” Journal of the National Cancer Institute 116, no. 5 (2024): 773. 40748540 10.1093/jnci/djae045

[2] R. L. Siegel , K. D. Miller , and A. Jemal , “Cancer Statistics, 2020,” CA: A Cancer Journal for Clinicians 70, no. 1 (2020): 7–30. 31912902 10.3322/caac.21590

[3] L. L. Page , T. P. Devasia , A. Mariotto , L. Gallicchio , M. A. Mollica , and E. Tonorezos , “Prevalence of Cancer Survivors Diagnosed During Adolescence and Young Adulthood in the United States,” Journal of the National Cancer Institute 117 (2024): 529–536. 10.1093/jnci/djae250 PMC11884855 39383200

[4] J. S. Wefel , S. R. Kesler , K. R. Noll , and S. B. Schagen , “Clinical Characteristics, Pathophysiology, and Management of Noncentral Nervous System Cancer‐Related Cognitive Impairment in Adults,” CA: A Cancer Journal for Clinicians 65, no. 2 (2015): 123–138. 25483452 10.3322/caac.21258 PMC4355212

[5] H. S. Jim , K. M. Phillips , S. Chait , et al., “Meta‐Analysis of Cognitive Functioning in Breast Cancer Survivors Previously Treated With Standard‐Dose Chemotherapy,” Journal of Clinical Oncology 30, no. 29 (2012): 3578–3587. 22927526 10.1200/JCO.2011.39.5640 PMC3462044

[6] S. J. Mayo , K. Edelstein , E. G. Atenafu , R. Ajaj , M. Li , and L. J. Bernstein , “Cognitive Symptoms Across Diverse Cancers,” JAMA Network Open 7, no. 8 (2024): e2430833. 39196555 10.1001/jamanetworkopen.2024.30833 PMC11358862

[7] H. S. L. Jim , S. L. Jennewein , G. P. Quinn , D. R. Reed , and B. J. Small , “Cognition in Adolescent and Young Adults Diagnosed With Cancer: An Understudied Problem,” Journal of Clinical Oncology 36, no. 27 (2018): 2752–2754. 30040524 10.1200/JCO.2018.78.0627 PMC7010417

[8] L. M. Vizer , S. P. Mikles , and A. T. Piepmeier , “Cancer‐Related Cognitive Impairment in Survivors of Adolescent and Young Adult Non‐Central Nervous System Cancer: A Scoping Review,” Psycho‐Oncology 31, no. 8 (2022): 1275–1285. 35726379 10.1002/pon.5980

[9] M. E. McGrady , V. W. Willard , A. M. Williams , and T. M. Brinkman , “Psychological Outcomes in Adolescent and Young Adult Cancer Survivors,” Journal of Clinical Oncology 42, no. 6 (2024): 707–716. 37967297 10.1200/JCO.23.01465 PMC13019686

[10] N. Boykoff , M. Moieni , and S. K. Subramanian , “Confronting Chemobrain: An In‐Depth Look at Survivors Reports of Impact on Work, Social Networks, and Health Care Response,” Journal of Cancer Survivorship: Research and Practice 3, no. 4 (2009): 223–232. 19760150 10.1007/s11764-009-0098-x PMC2775113

[11] S. K. Parsons , T. H. M. Keegan , A. C. Kirchhoff , H. M. Parsons , K. R. Yabroff , and S. J. Davies , “Cost of Cancer in Adolescents and Young Adults in the United States: Results of the 2021 Report by Deloitte Access Economics, Commissioned by Teen Cancer America,” Journal of Clinical Oncology 41, no. 17 (2023): 3260–3268. 36827624 10.1200/JCO.22.01985 PMC10256335

[12] B. Thom , D. N. Friedman , E. M. Aviki , et al., “The Long‐Term Financial Experiences of Adolescent and Young Adult Cancer Survivors,” Journal of Cancer Survivorship 17, no. 6 (2023): 1813–1823. 36472761 10.1007/s11764-022-01280-2 PMC9734817

[13] J. K. Hartshorne and L. T. Germine , “When Does Cognitive Functioning Peak ‐ The Asynchronous Rise and Fall of Different Cognitive Abilities Across the Life Span,” Psychological Science 26, no. 4 (2015): 433–443. 25770099 10.1177/0956797614567339 PMC4441622

[14] J. J. McArdle , K. J. Grimm , F. Hamagami , R. P. Bowles , and W. Meredith , “Modeling Life‐Span Growth Curves of Cognition Using Longitudinal Data With Multiple Samples and Changing Scales of Measurement,” Psychological Methods 14, no. 2 (2009): 126–149. 19485625 10.1037/a0015857 PMC2831479

[15] A. Chan , I. Cheng , C. Wang , et al., “Cognitive Impairment in Adolescent and Young Adult Cancer Patients: Pre‐Treatment Findings of a Longitudinal Study,” Cancer Medicine 12, no. 4 (2023): 4821–4831. 36221816 10.1002/cam4.5295 PMC9972136

[16] E. O. Dewar , C. Ahn , S. Eraj , B. A. Mahal , and N. N. Sanford , “Psychological Distress and Cognition Among Long‐Term Survivors of Adolescent and Young Adult Cancer in the USA,” Journal of Cancer Survivorship: Research and Practice 15, no. 5 (2021): 776–784. 33415652 10.1007/s11764-020-00969-6

[17] D. B. Tometich , T. Welniak , L. M. Gudenkauf , et al., “I Couldnot Connect the Wires in My Brain, Young adult cancer survivors experience with cognitive functioning,” Psycho‐Oncology 33 (2024): e6309. 38420860 10.1002/pon.6309 PMC11249037

[18] D. S. J. Costa and J. E. Fardell , “Why Are Objective and Perceived Cognitive Function Weakly Correlated in Patients With Cancer?,” Journal of Clinical Oncology 37, no. 14 (2019): 1154–1158. 30920881 10.1200/JCO.18.02363

[19] A. M. Henneghan , K. Van Dyk , T. Kaufmann , et al., “Measuring Self‐Reported Cancer‐Related Cognitive Impairment: Recommendations From the Cancer Neuroscience Initiative Working Group,” Journal of the National Cancer Institute 113, no. 12 (2021): 1625–1633. 33638633 10.1093/jnci/djab027 PMC8849125

[20] B. M. Veal , S. B. Scott , H. S. L. Jim , and B. J. Small , “Subjective Cognition and Memory Lapses in the Daily Lives of Breast Cancer Survivors: Examining Associations With Objective Cognitive Performance, Fatigue, and Depressed Mood,” Psycho‐Oncology 32, no. 8 (2023): 1298–1305. 37381150 10.1002/pon.6185 PMC10859854

[21] T. W. Robbins , M. James , A. M. Owen , B. J. Sahakian , L. McInnes , and P. Rabbitt , “Cambridge Neuropsychological Test Automated Battery (CANTAB): A Factor Analytic Study of a Large Sample of Normal Elderly Volunteers,” Dementia 5, no. 5 (1994): 266–281. 7951684 10.1159/000106735

[22] A. Egerhazi , R. Berecz , E. Bartok , and I. Degrell , “Automated Neuropsychological Test Battery (CANTAB) in Mild Cognitive Impairment and in Alzheimers Disease,” Progress in Neuro‐Psychopharmacology & Biological Psychiatry 31, no. 3 (2007): 746–751. 17289240 10.1016/j.pnpbp.2007.01.011

[23] C. M. Bender , J. D. Merriman , A. L. Gentry , et al., “Patterns of Change in Cognitive Function With Anastrozole Therapy,” Cancer 121, no. 15 (2015): 2627–2636. 25906766 10.1002/cncr.29393 PMC4512875

[24] L. Capuron , A. Ravaud , and R. Dantzer , “Timing and Specificity of the Cognitive Changes Induced by Interleukin‐2 and Interferon‐Alpha Treatments in Cancer Patients,” Psychosomatic Medicine 63, no. 3 (2001): 376–386. 11382265 10.1097/00006842-200105000-00007

[25] J. L. Vardy , H. M. Dhillon , G. R. Pond , et al., “Cognitive Function in Patients With Colorectal Cancer Who Do and Do Not Receive Chemotherapy: A Prospective, Longitudinal, Controlled Study,” Journal of Clinical Oncology 33, no. 34 (2015): 4085–4092. 26527785 10.1200/JCO.2015.63.0905 PMC5683012

[26] H. S. Jim , K. A. Donovan , B. J. Small , M. A. Andrykowski , P. N. Munster , and P. B. Jacobsen , “Cognitive Functioning in Breast Cancer Survivors: A Controlled Comparison,” Cancer 115, no. 8 (2009): 1776–1783. 19224550 10.1002/cncr.24192 PMC2668740

[27] K. M. Phillips , H. S. Jim , B. J. Small , C. Laronga , M. A. Andrykowski , and P. B. Jacobsen , “Cognitive Functioning After Cancer Treatment: A 3‐Year Longitudinal Comparison of Breast Cancer Survivors Treated With Chemotherapy or Radiation and Noncancer Controls,” Cancer 118, no. 7 (2012): 1925–1932. 22161750 10.1002/cncr.26432 PMC3305821

[28] B. J. Small , K. S. Rawson , E. Walsh , et al., “Catechol‐O‐Methyltransferase Genotype Modulates Cancer Treatment‐Related Cognitive Deficits in Breast Cancer Survivors,” Cancer 117, no. 7 (2011): 1369–1376. 21425136 10.1002/cncr.25685

[29] Limited CC , CANTAB Connect Research: Admin Application User Guide (Cambridge Cognition, 2022).

[30] R. Fieo , K. Ocepek‐Welikson , M. Kleinman , et al., “Measurement Equivalence of the Patient Reported Outcomes Measurement Information System((R)) (PROMIS((R))) Applied Cognition, General Concerns, Short Forms in Ethnically Diverse Groups,” Psychological Test and Assessment Modeling 58, no. 2 (2016): 255–307. 28523238 PMC5433382

[31] J. M. Salsman , S. C. Danhauer , J. B. Moore , et al., “Optimizing the Measurement of Health‐Related Quality of Life in Adolescents and Young Adults With Cancer,” Cancer 126, no. 22 (2020): 4818–4824. 32910454 10.1002/cncr.33155 PMC8005324

[32] D. Cella , W. Riley , A. Stone , et al., “The Patient‐Reported Outcomes Measurement Information System (PROMIS) Developed and Tested Its First Wave of Adult Self‐Reported Health Outcome Item Banks: 2005‐2008,” Journal of Clinical Epidemiology 63, no. 11 (2010): 1179–1194. 20685078 10.1016/j.jclinepi.2010.04.011 PMC2965562

[33] J. M. Cessna , H. S. Jim , S. K. Sutton , et al., “Evaluation of the Psychometric Properties of the PROMIS Cancer Fatigue Short Form With Cancer Patients,” Journal of Psychosomatic Research 81 (2016): 9–13. 26800633 10.1016/j.jpsychores.2015.12.002 PMC4822706

[34] A. A. Stone , J. E. Broderick , D. U. Junghaenel , S. Schneider , and J. E. Schwartz , “PROMIS Fatigue, Pain Intensity, Pain Interference, Pain Behavior, Physical Function, Depression, Anxiety, and Anger Scales Demonstrate Ecological Validity,” Journal of Clinical Epidemiology 74 (2016): 194–206. 26628334 10.1016/j.jclinepi.2015.08.029

[35] Y. W. Leung , C. Brown , A. P. Cosio , et al., “Feasibility and Diagnostic Accuracy of the Patient‐Reported Outcomes Measurement Information System (PROMIS) Item Banks for Routine Surveillance of Sleep and Fatigue Problems in Ambulatory Cancer Care,” Cancer 122, no. 18 (2016): 2906–2917. 27351521 10.1002/cncr.30134

[36] R. J. Ferguson , B. C. McDonald , A. J. Saykin , and T. A. Ahles , “Brain Structure and Function Differences in Monozygotic Twins: Possible Effects of Breast Cancer Chemotherapy,” Journal of Clinical Oncology 25, no. 25 (2007): 3866–3870. 17761972 10.1200/JCO.2007.10.8639 PMC3329758

[37] B. J. Small , H. S. L. Jim , S. L. Eisel , P. B. Jacobsen , and S. B. Scott , “Cognitive Performance of Breast Cancer Survivors in Daily Life: Role of Fatigue and Depressed Mood,” Psycho‐Oncology 28, no. 11 (2019): 2174–2180. 31418499 10.1002/pon.5203 PMC6858929

[38] S. B. Scott , J. A. Mogle , M. J. Sliwinski , H. S. L. Jim , and B. J. Small , “Memory Lapses in Daily Life Among Breast Cancer Survivors and Women Without Cancer History,” Psycho‐Oncology 29, no. 5 (2020): 861–868. 32040229 10.1002/pon.5357 PMC10141683

[39] V. Osmani , L. Hörner , S. J. Klug , and L. F. Tanaka , “Prevalence and Risk of Psychological Distress, Anxiety and Depression in Adolescent and Young Adult (AYA) Cancer Survivors: A Systematic Review and Meta‐Analysis,” Cancer Medicine 12, no. 17 (2023): 18354–18367. 37559504 10.1002/cam4.6435 PMC10523984

[40] P. W. Dankers , S. H. M. Janssen , M. van Eenbergen , B. M. Siflinger , W. T. A. van der Graaf , and O. Husson , “Employment Outcomes of Adolescent and Young Adult Cancer Survivors and Their Partners: A Dutch Population‐Based Study,” Cancer 130, no. 13 (2024): 2372–2383. 38396253 10.1002/cncr.35260

