# Supplementary material for: Characterizing Cancer‐Related Cognitive Impairment Among Adolescent and Young Adult Cancer Survivors: Comparison to Individuals Without Cancer
Source: Cancer Med. 2025 Nov 5;14(21):e71363. doi: 10.1002/cam4.71363 (PMC12588723; doi:10.1002/cam4.71363)
Supplement: Supplementary file 1 — Table S1: Description of the CANTAB test domains and individual tests. Table S2: Group differences in CANTAB objective cognitive performance. [file CAM4-14-e71363-s001.docx]

Supplemental Table 1. Description of the CANTAB Test Domains and Individual Tests

Domain/Measure Description

**Attention/Rapid Visual Information Processing**

A’ Ranging from 0 (bad) to 1 (good), this is a metric of how well the participant is at detecting target sequences.

Median response latency (msec) Median response latency on trials where the participant responded correctly

**Attention/Delayed Match to Sample**

% correct – All Delays Percentage of assessment trials where participant selected the correct box on their first choice, calculated across all trials that contained a delay

% correct – Simultaneous display Percentage of assessment trials where participant selected the correct box on their first choice, calculated across trials where the target and response was presented simultaneously.

% correct – 0 second delay Percentage of assessment trials where participant selected the correct box on their first choice, calculated across trials containing a 0 second delay.

% correct – 4 second delay Percentage of assessment trials where participant selected the correct box on their first choice, calculated across trials containing a 4 second delay.

% correct – 12 second delay Percentage of assessment trials where participant selected the correct box on their first choice, calculated across trials containing a 12 second delay.

**Executive Functioning/Spatial Span**

Span length – forward sequence Longest sequence of boxes successfully recalled by participants in the forward sequence.

Span length – reverse sequence Longest sequence of boxes successfully recalled by participants in the forward sequence.

**Memory/Paired Associate Learning**

First memory attempt score Number of times that a participant chose the correct box on their first attempt when recalling the pattern locations.

Total Errors Number of times that a participant chose the incorrect box when recalling the pattern locations.

**Memory/Spatial Working Memory**

Strategy (6-8 boxes) A measure of the extent to which participant employs a planned strategy by beginning search from the same starting point.

Errors Number of times a participant revisits a box in which the target was previously found.

Source: CANTAB Connect Research: Admin Application User Guide. Cambridge, UK.

Supplemental Table 2: Group differences in CANTAB objective cognitive performance

AYA Non-Cancer Effect

Survivors Controls Size (d) p

**Attention-Rapid Visual Information Processing (AYA n = 74; NC n = 90)**

A’ M .90 .91 0.11 .721

SE .01 .01

Median response M 479.54 442.69 0.26 .114

latency (msec) SE 16.48 14.80

**Attention – Delayed Match to Sample (AYA n = 70; NC n – 90)**

% correct – All Delays M 84.87 88.09 0.25 .129

SE 1.52 1.33

% correct - Simultaneous M 98.86 98.22 0.12 .486

display SE 0.67 0.58

% correct – 0 second M 87.65 90.05 0.14 .405

delay SE 2.07 1.81

% correct – 4 second M 83.69 89.13 0.31 .069

delay SE 2.15 1.87

% correct – 12 second M 83.37 85.16 0.09 .592

delay SE 2.39 2.09

**Executive Functioning – Spatial Span (AYA n = 71; NC n = 87)**

Span length – forward M 7.31 7.08 0.17 .338

sequence SE .16 .15

Span length - reverse M 6.54 6.92 0.23 .191

sequence SE 0.20 0.18

**Memory – Paired Associate Learning (AYA n = 74; NC n = 90)**

First memory attempt M 14.62 15.99 0.36 .032

score SE .45 .40

Total Errors M 8.63 6.26 0.26 .117

SE 1.07 .96

**Memory - Spatial Working Memory (AYA n = 75; NC n = 90)**

Strategy (6-8 boxes) M 6.14 6.48 0.12 .491

SE 0.34 0.31

Errors M 7.25 6.75 0.06 .706

SE 0.95 0.86
